# Inhalation

**Published:** 1880-11

**Authors:** 


### INHALATION AND CONSUMPTION.

My own views are, that no medicine known has any direct curative effect in consumptive diseases; that the only cure is in the perfect action of the *Chylopoietic Viscera*, that is, of the food digesting and blood making organs, the chief of which are the stomach, liver and bowels.

Consumption is literally *defective nutrition:* the whole man dwindles; flesh, strength, breath, all waste away by painfully slow degrees; *not the lungs alone*, which make but a small part of what is really affected, the whole machinery of the man is in disorder. The patient may eat heartily to the last day almost, yet the system has not the power to extract from the food eaten, and properly appropriate, the nutriment which it naturally possesses; hence the uniform remark "I eat well, but it does not seem to strengthen me." In almost all other diseases, the ability and appetite to eat is followed very shortly with increasing flesh and bodily vigor. How then can medicinal substances applied to the lungs, have any really curative effect, when it is in the stomach, liver and bowels, where the very foundation of the ailment lies? for it is there the blood is made, out of which the tubercle, the seed of consumption is formed, as all allow. The blood is not made consumptive in the lungs, but carries consumption with it to the lungs in its imperfect elimination, in its imperfectness of material, which material is drawn from the food after the stomach has acted upon it. And all that medicine can do towards curing consumption, is by the aid which it affords the stomach, liver and bowels in calling for, in receiving and in digesting the food which we swallow. Whatever gives appetite, and with it an increase of digestive power, that is radically curative of consumption. Any man who can be cured at all, will be cured by taking into his lungs the largest amount of out door air, and by imposing upon his body the largest amount of muscular exercise, not involving actual fatigue, the most favorable combination of all which, is found in continuous, active, horseback exercise on the open road, from morning till night, from one week's end to another, with some pleasurable and profitably absorbing object ahead, other than the mere health; in all of which, being under the care of some *one*, honorable, educated physician who possesses your confidence and respect. This, reader, is the embodied result, without any mental reservation whatever, of the observation and experience of a young life time, spent in the treatment and study of this one disease, and upon its truth, I, with steady confidence, do stake my reputation and my daily bread. But why is active exercise on horseback on the open road so largely insisted on? simply because in the experience of all, nothing else is so effectual in giving a good appetite, and securing a good digestion; the exercise giving the digestion without the over fatigue attendant on bodily labor, and every breath drawn being a breath of pure air, unloads the blood of its impurities in the lungs, to be sent thence to the most distant portion of the human body at the rate of a hogshead an hour; imparting life and energy to every fibre.

What then the need of a physician? To know *first* the actual condition of things and to meet incidental and occasional symptoms promptly, before they gain power. An infant's arm may stop an avalanche, a moment later and millions of men are powerless. And then again, comparatively few have it in their power to carry out the practice indicated, at least to its full extent; then, the judgment, tact and experience of the physician are to be brought into requisition to provide substitutes, medicinal and otherwise.

It is not contended that there are no benefits whatever to be derived from breathing air saturated with something else, whether drugs, liquors, aliments or other things, but we do contend that such effects are transient, are not radically curative, but mere alleviants, only giving time for other more efficient means to be brought to bear. We tried the whole thing years ago and found no worthy result in any single instance. But say these new men, "that was because you did not use the right material and in a proper manner, but we have found out a new inhalent, and hence our success."

